# Supplementary material for: A case-control study evaluating CT signs of xiphoid process associated with xiphodynia
Source: PLoS One. 2025 May 20;20(5):e0303657. doi: 10.1371/journal.pone.0303657 (PMC12091747; doi:10.1371/journal.pone.0303657)
Supplement: Attachment — Submitted filename: Response to Reviewers.docx [file pone.0303657.s003.docx]

Dear Reviewers,

I would like to express my sincere gratitude for the thorough review and constructive feedback you provided on my manuscript submitted to PLOS ONE. Your insightful comments have been invaluable in improving the quality and clarity of the manuscript.

I have carefully considered each of your suggestions and incorporated them into the revised version. Your expertise and thoughtful input have significantly contributed to the overall enhancement of the paper.

Thank you once again for your time, effort, and valuable contributions to the advancement of this work. Your dedication to the peer review process is greatly appreciated.

Best regards,

Corresponding Author: Ryosuke Ono

Department of Community Medicine, Kameyama

[rikusan2005@yahoo.co.jp](mailto:rikusan2005@yahoo.co.jp)

Journal Requirements:

1. When submitting your revision, we need you to address these additional requirements. Please ensure that your manuscript meets PLOS ONE's style requirements, including those for file naming. The PLOS ONE style templates can be found at

<https://journals.plos.org/plosone/s/file?id=wjVg/PLOSOne_formatting_sample_main_body.pdf> and

<https://journals.plos.org/plosone/s/file?id=ba62/PLOSOne_formatting_sample_title_authors_affiliations.pdf>

**→We have confirmed that the submitted file meets PLOS ONE's style requirements.**

2. Thank you for uploading your study's underlying data set. Unfortunately, the repository you have noted in your Data Availability statement does not qualify as an acceptable data repository according to PLOS's standards. At this time, please upload the minimal data set necessary to replicate your study's findings to a stable, public repository (such as figshare or Dryad) and provide us with the relevant URLs, DOIs, or accession numbers that may be used to access these data. For a list of recommended repositories and additional information on PLOS standards for data deposition, please see <https://journals.plos.org/plosone/s/recommended-repositories>.

3. In the online submission form, you indicated that your data will be submitted to a repository upon acceptance. We strongly recommend all authors deposit their data before acceptance, as the process can be lengthy and hold up publication timelines. Please note that, though access restrictions are acceptable now, your entire minimal dataset will need to be made freely accessible if your manuscript is accepted for publication. This policy applies to all data except where public deposition would breach compliance with the protocol approved by your research ethics board. If you are unable to adhere to our open data policy, please kindly revise your statement to explain your reasoning and we will seek the editor's input on an exemption.

**→We have uploaded the data to Dryad. Below are the Reviewer link and DOI for your reference.**

ReviewerURL: <https://datadryad.org/stash/share/12RMAxde1Qt6QDs9SCraPdbrGHPpKrE1-DYXcfPtJxU>

doi:10.5061/dryad.ghx3ffbxw

4. Please review your reference list to ensure that it is complete and correct. If you have cited papers that have been retracted, please include the rationale for doing so in the manuscript text, or remove these references and replace them with relevant current references. Any changes to the reference list should be mentioned in the rebuttal letter that accompanies your revised manuscript. If you need to cite a retracted article, indicate the article’s retracted status in the References list and also include a citation and full reference for the retraction notice.

**→We have confirmed that all references are publicly accessible. References 2 and 15 are not indexed in PubMed.**

Reviewer #1: This is an interesting study of the xiphoid process, has been evaluated by CT scan, in the diagnosing xiphodynia. concluded that The xiphoid process sternal angle is not useful for diagnosing xiphodynia. The paper is well written and ethical issues are considered appropriately.

**→Thank you for your evaluation. It has become an encouragement for my future research career.**

Reviewer #2: First, I would like to congratulate the authors on an interesting study and the efforts put forth to answer a rare clinical conundrum. Overall, the manuscript is well written, easy to read, and understand. There are a few minor syntax errors that could be corrected. In addition, would consider using the term study group rather than case group in the abstract as it reads better.

**→Thank you very much for your feedback. We have made some corrections based on it.**

In some instance, you may want to consider using the term xiphosternal angulation as oppose to angle as this represents a phenomenon rather than an anatomic finding.

**→In the discussion, we changed "angle" to "angulation" in two instances where it conveyed the meaning of "a phenomenon rather than an anatomic finding." Upon further investigation into the meanings of "angulation" and "angle," as you pointed out, it seems that "xiphisternal angulation" would convey a more appropriate nuance than "xiphisternal angle." However, since "xiphisternal angle" has been consistently used in previous literatures, we have opted to adopt this term in our study as well.**

In lines 169, 171, and 173, recommend specification of the origin of the line, for example, "from the anterior abdominal wall to..." as any line has to have two points of reference.

**→I am debating whether to make the changes as you suggested. For the B-line, I considered modifying it from "A tangent line to the midline of the base of the xiphoid process" to "A line perpendicular to the midpoint between the ventral and dorsal sides at the base of the xiphoid process." However, I believe this expression would not be suitable for the D-line because the thickness at the tip of the xiphoid process is approximately zero. Moreover, considering that tangency is defined as "a straight line passing through two points infinitely close on a curve," it seems unnecessary to strictly define two points.**

**Ultimately, the key is how to define these lines to enhance the reproducibility of the study. Since we initially adopted tangents as curves rather than midlines of thickness, we prefer to use the original description. If changes are still preferable based on the considerations above, I am willing to make adjustments. Your feedback on this matter would be appreciated.**

In the table on page 10, please reduce the size of the table 2 to conform to the page as it runs over the margin.

**→We have made adjustments to fit within the framework.**

In line 359 and 360 on page 13, the phrase is not clear, "although not explicitly stated the angles?" Please revise, perhaps, "although not explicitly stated regarding the angles..."

**→As you suggested, we have made the changes.**
