# Supplementary material for: A case-control study evaluating CT signs of xiphoid process associated with xiphodynia
Source: PLoS One. 2025 May 20;20(5):e0303657. doi: 10.1371/journal.pone.0303657 (PMC12091747; doi:10.1371/journal.pone.0303657)
Supplement: Attachment — Submitted filename: Response_to_Reviewers_auresp_2.docx [file pone.0303657.s004.docx]

Dear Editors and Reviewers,

I am writing to submit the revised version of my manuscript titled “[Title of Your Manuscript]” for reconsideration by PLOS ONE. I sincerely appreciate the additional feedback and comments provided by the reviewers and editorial team following my previous submission. Their continued guidance has been instrumental in refining the manuscript further.

In this revision, I have made every effort to ensure that the manuscript adheres to the submission guidelines of PLOS ONE. Notably, the tables have been converted to figures in accordance with the journal’s requirements. I hope that my submission is now fully compliant with the guidelines.

Thank you once again for your time, effort, and valuable input in reviewing this work. I look forward to your feedback on the revised manuscript.

Best regards,

Corresponding Author: Ryosuke Ono

Department of Community Medicine, Kameyama

[rikusan2005@yahoo.co.jp](mailto:rikusan2005@yahoo.co.jp)

Journal Requirements:
Please review your reference list to ensure that it is complete and correct. If you have cited papers that have been retracted, please include the rationale for doing so in the manuscript text, or remove these references and replace them with relevant current references. Any changes to the reference list should be mentioned in the rebuttal letter that accompanies your revised manuscript. If you need to cite a retracted article, indicate the article’s retracted status in the References list and also include a citation and full reference for the retraction notice.
**→We have corrected the author names in reference 23, which were previously in uppercase.**

Additional Editor Comments:
Thanks for addressing all the reviewer comments. Please make sure that your manuscript structure follows the PLOS ONE formatting sample.
Please submit an unstructured abstract.
revise your references, figures, and tables to follow the PLOS ONE formatting sample (find the attached PDF)

**→We have revised the abstract to make it less structured. Additionally, we have converted all tables to figures.** **The font in the figures has been changed to the specified one, and PACE has also been used.**

Reviewer #2: Recommend changing the last reference to the appropriate case as the authors names do not need to be in all capitalized letter.

**→We hadn't noticed this until it was pointed out, so we appreciate your help. We have made the necessary corrections.**
